# Spontaneous idiopathic spinal subdural hemorrhage in a 16‐year‐old boy: A rare case in pediatrics and review of literature

**DOI:** 10.1002/ccr3.6993

**Published:** 2023-02-24

**Authors:** Iman Ahrari, Mohamad Jamali, Somayeh Mohammadi, Mahsa Ghavipisheh, Keyvan Eghbal, Arash Saffarian, Abdolkarim Rahmanian, Soulmaz Ghahramani, Reza Taheri

**Affiliations:** ^1^ Department of Neurosurgery Shiraz University of Medial Sciences Shiraz Iran; ^2^ Department of Anesthesiology, Preoperative and Pain Medicine, Brigham and Woman's Hospital Harvard Medical School Boston MA USA; ^3^ Department of Psychiatry Shiraz University of Medial Sciences Shiraz Iran; ^4^ Health Policy Research Center, Institute of Health Shiraz University of Medical Sciences Shiraz Iran

**Keywords:** cervical spine, idiopathic, pediatric, spontaneous subdural hematoma, subdural hematoma

## Abstract

Idiopathic spinal subdural hematoma (SSDH) is a rare phenomenon. Here, we present a 16‐year‐old‐boy who presented with acute sudden onset weakness and brown squared syndrome; the cervical MRI findings showed acute subdural hematoma from C2 to C6. Emergent surgical intervention was performed, and significant improvement was seen in follow‐ups.

## Abstract 2

Spontaneous spinal subdural hematoma is a rare phenomenon, particularly in pediatrics. The thoracic spine is the most prevalent area. Back pain and extremities weakness are common findings. Emergent surgical intervention would have favorable outcomes.

## 1 INTRODUCTION

Spontaneous subdural hematoma (SSDH) is a rare condition resulting in cord compression and is associated with significant mortality and morbidity. Spinal SDH can be secondary to anticoagulation therapy, blood dyscrasia, spinal puncture, trauma, spinal anesthesia, or vascular malformation. However, spontaneous SDH is rare, and pathophysiology is still unknown.^1^

Rupture of the vasculature within the subarachnoid or subdural space has been proposed as a potential pathogenic mechanism in certain cases. While some suggest that the bleeding originates from the subarachnoid vessels with concomitant rupture into the subdural space following an increase in intra‐abdominal or intra‐thoracic pressure, others have proposed an alternative theory that the bleeding begins in the subdural space itself.^2^

The clinical manifestations of SSDH are related to cord compression and vary from back pain to motor, sensory, and autonomic dysfunction.^3^, ^4^ The main approach for confirming the diagnosis is magnetic resonance imaging (MRI).^5^ Although surgical intervention through decompression is considered as the main treatment option, percutaneous drainage or conservative therapies are also introduced. There is still controversy about the best therapeutic strategy.^4^

In this report, we present a case of cervical SSDH who presented with acute left side weakness with no identifiable cause.

## 2 CASE REPORT

A 16‐year‐old boy presented with sudden onset, rapid progressive weakness of the left side extremities. In the examination, the patient had right side hypoesthesia to pain and light touch (positive brown squared syndrome). No urinary or fecal incontinency was detected in the physical examination. The patient had no positive history for any recent trauma, and his past medical history was negative for any hematologic disease. Laboratory examinations did not show any coagulopathy or blood dyscrasia.

**FIGURE 1:**
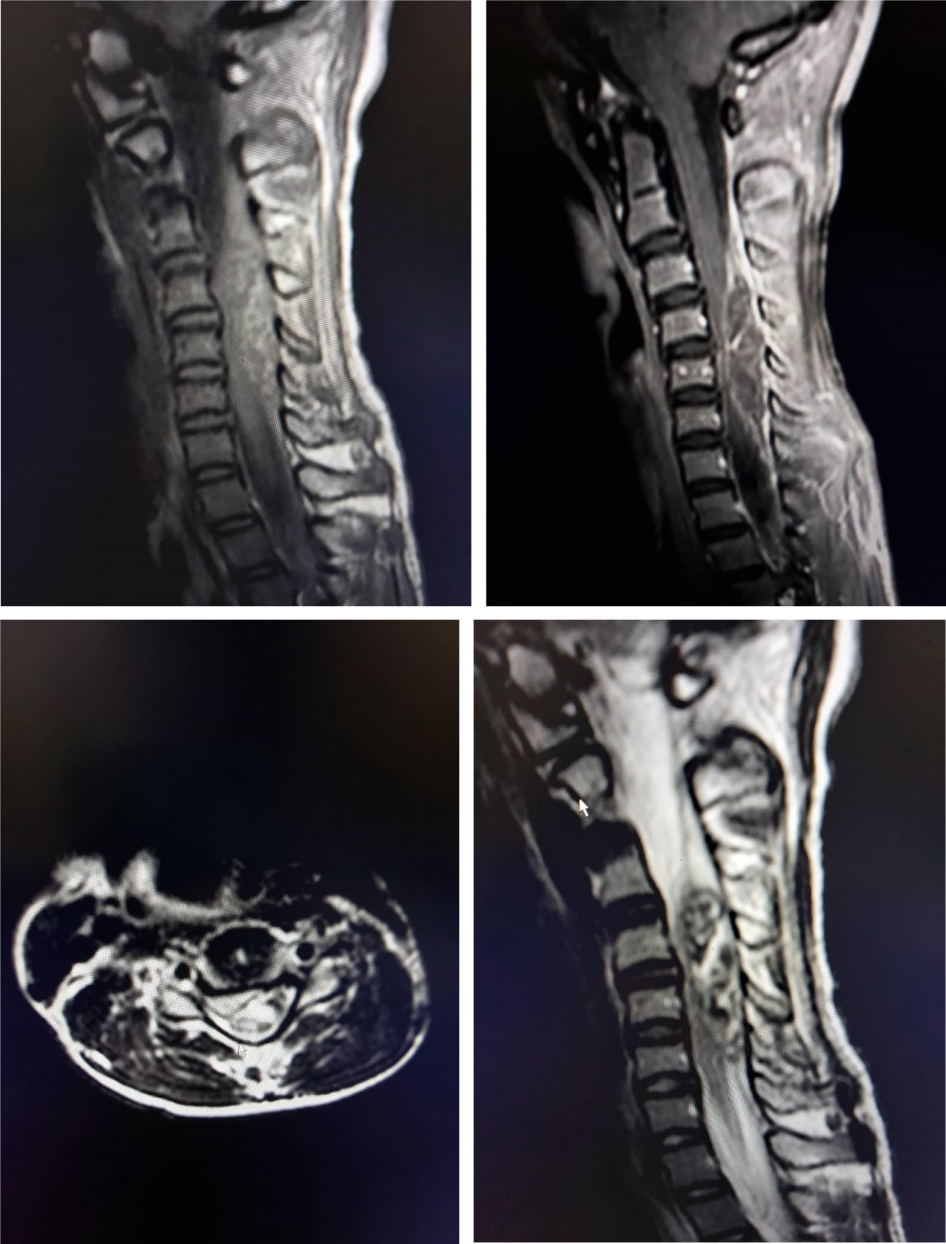
Magnetic resonance imaging of the patient with spontaneous idiopathic spinal subdural hemorrhage; in sagittal T1 images, mixed and hyper‐signal intensity lesion and in T2 images mixed and hypo‐signal lesion are seen. After gadolinium injection, only mild peripheral enhancement was seen. In axial T2 images, the lesion is located posterolateral to the spinal cord in the left side.

Preoperative MRI was done, showing a heterogeneous extra‐medullary lesion posterolateral to the cord, spanning from C2 to C6 (Figure 1) with hyper‐intensity in T1‐weighted images, hypo‐intensity in T2‐weighted images, and without significant enhancement after gadolinium injection. Significant compression was made on the cord roots in the left side.

**FIGURE 2:**
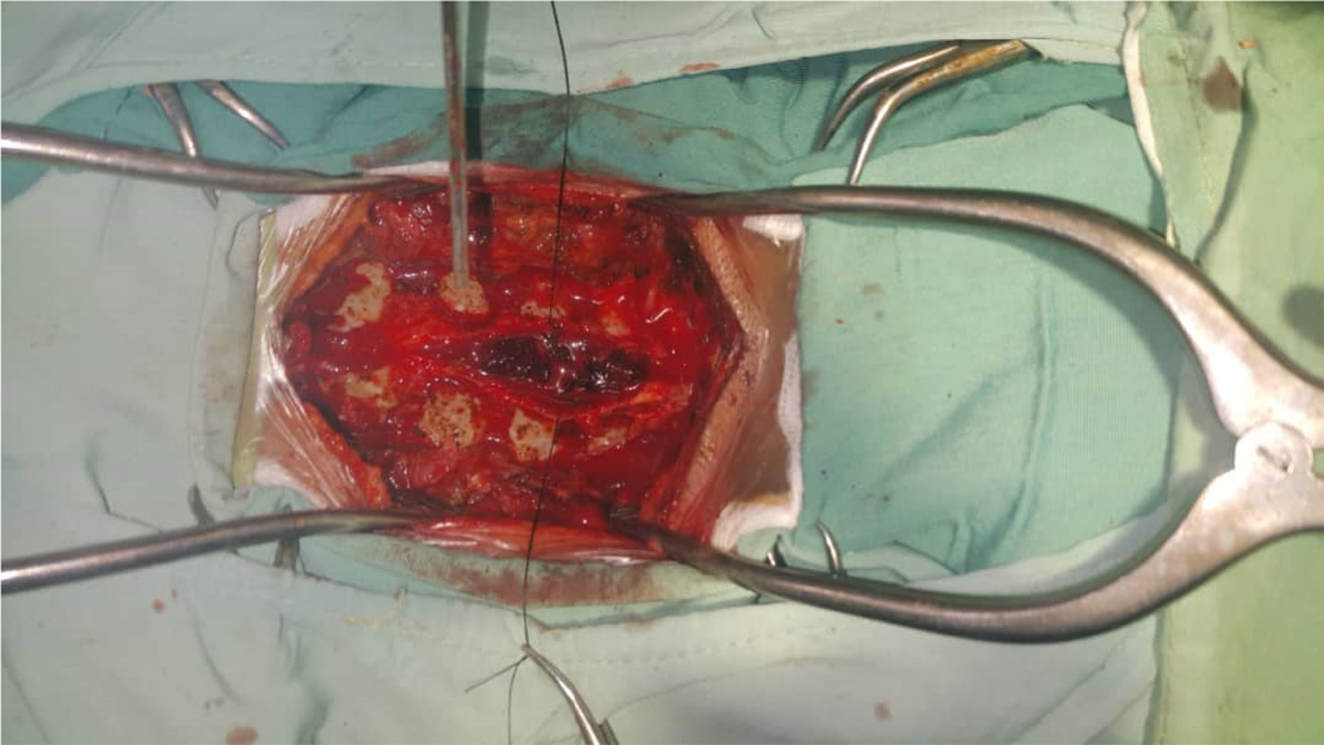
Intra‐operative view; after laminectomy and dural opening, the hematoma was evacuated.

The patient was transferred to the operation room and underwent emergency decompression by C3–C7 conservative laminectomy. The dura was opened, and subdural hemorrhage was evacuated. During the operation, a hematoma was visualized upon the opening of the thecal sac and evacuated with gentle suction (Figure 2). No evidence of abnormal vasculature or masses was observed. Hematoma fragments were collected and sent for histo‐pathologic evaluation. Pathologic report of the lesion confirmed the diagnosis of hematoma. The patient underwent magnetic resonant angiography (MRA) post‐op for detection of vascular disorders, but it did not show any abnormality.

In the post‐operation examination, the cervical pain was completely healed and two days post‐operation the weakness in the extremities was gradually improved. Left side extremity strength improved to consistent grade 2/5 throughout with reported rare ability to move his leg against gravity. Four weeks after surgery in outpatient follow‐up, motor power showed a remarkable improvement, and he could walk on his own feet.

## 3 REVIEW OF THE LITERATURE

A review of the English literature was conducted by searching Medline and PubMed from 2010 to August 2021. The terms “spinal subdural hematoma,” “acute spinal subdural hematoma,” “spontaneous spinal subdural hematoma,” and “idiopathic spinal subdural hematoma” were used. In a search of Medline, totally 42 articles were found. The papers were reviewed; if the history of trauma, coagulopathy, vascular abnormality, or iatrogenic cause was found, the papers were excluded. The studied factors were age, gender, sign and symptoms, level of involvement, type of treatment (surgical intervention or conservative therapy), and patient outcome. After exclusion of unrelated articles, 20 papers were included in our study containing the present case^1^, ^6^, ^7^, ^8^, ^9^, ^10^, ^11^, ^12^, ^13^, ^14^, ^15^, ^16^, ^17^, ^18^, ^19^, ^20^ (Table 1).

**TABLE 1:** Summary of the results from the literature review: cases of spontaneous spinal subdural hematoma.

Author and year	Age, years	Sex	Location	Presenting symptoms	Potential RFs	Treatment	Outcome
Cave and Sharobeem, 2013^1^	65	M	T12	Back pain, paraplegia	No	Conservative	Partial recovery
Chung et al., 2014^2^	66	F	C7–T4	HA, neck stiffness	No	Conservative	Complete recovery
Cui et al., 2015^3^	45	M	L4–S3	Saddle pain	No	Laminectomy	Good recovery
de Beer MH et al., 2017^4^	Adult		T3–T12	Paraplegia	HTN	Laminectomy	Good recovery
de Beer MH et al., 2017^4^	Adult		T11	Pain and paraparesis	HTN	Laminectomy	Good recovery
Kakitsubata et al., 2010^6^	66	M	T11‐T12	HA, back pain,L LE pain	No	Conservative	Complete recovery
Lin and Layman, 2014^8^	70	M	L4–S1	Back pain, BLE weakness	HTN, hyperlipidemia, cancer	Conservative	Partial recovery
Liu et al., 2010^9^	41	M	Mid T spine	Back pain, par paresis, bladder dysfunction	Rhabdomyolysis, amphetamine abuse	Laminectomy T10‐L1	Good recovery
Ma et al., 2015^10^	29	F	C6–T2	Neck pain, para paresis	No	Conservative	Improvement
Nagashima et al., 2010^12^	66	M	L1–S1	Leg pain, paraparesis, hypoesthesia, bowel dysfunction	Concurrent intracranial SDH, RA, HTN	Conservative	Complete recovery
Nardone et al., 2010^13^	37	M	C4–T4	HA, neck stiffness, cervical radicular pain, paraparesis, hypoesthesia	No	Conservative	Complete recovery
Oh and Eun, 2015^14^	27	M	T5–T9	Back pain, paraparesis, hypoesthesia, bow dysfunction, erectile dysfunction	No	Conservative	Good recovery
Panciani et al., 2013^15^	79	F	C5–T6	Para paresis, urinary retention	No	Conservative	Improvement
Rettenmaier LA.2017^16^	43	M	T8–T11	Back pain, paraplegia	Drug abuse	Laminectomy	Poor recovery
Song et al., 2011^17^	57	M	C1–T3	Neck and shoulder pain, paraparesis	Chronic renal failure, HTN	Conservative	Complete recovery
Visocchi et al., 2015^18^	45	F	T1–T10	Back pain, paraplegia, anesthesia, bladder, and bowl dysfunction	HIV+, HCV+, history of drug abuse	Laminectomy	Partial recovery
Yang et al., 2011^20^	55	F	C2–T6	Paraplegia, hypoesthesia	HTN, DM	Conservative	Good recovery
Yang et al., 2011^20^	38	M	C6–T5	HA, back pain, cold sweating, dizziness, vertigo, chest pain, hypoesthesia	No	Conservative	Good recovery
Zhu et al., 2015^21^	45	F	T9	Paraplegia, hypoesthesia	No	Laminectomy T8–T10	Partial recovery
Present case	16	M	C2–C6	Hemiparesis and brown squared	No	Laminectomy	Good recovery

To the best of our knowledge, this is the first case of acute nontraumatic idiopathic SSDH in pediatric age patients considering it is very rare in pediatrics, which presented with acute paresthesia and weakness. Previously, 20 cases were reported in the literature on spontaneous spinal SDH from 2010. Of these cases, 12 were male and 7 were female. The patients' age ranged from 27 to 79 years with a mean of 49 years; only 8% were under 30, and one patient was ≤ 27. In most of the cases (80%), thoracic and cervicothoracic areas were involved; also, the lumbosacral area was involved in 14% of cases, and in only one case (present case), cervical segment was involved. The most common presentation was acute paresthesia and back‐pain. Other common symptoms consist of bladder, bowl dysfunction, and paraplegia. Laminectomy was done as the main surgical intervention in eight cases and conservative therapy in 13 cases. Seventeen cases experienced good recovery, three of whom had partial recovery, and only one case had poor recovery. The percentage of good recovery in those who underwent surgery was 62.5%, while this percentage was 84.7 in the conservative treatment group.

Our case was the first report of SSDH in pediatric and the third case of cervical subdural hematoma. The outcome of spontaneous subdural hematoma in the spine seems to be favorable. About 90% of cases had partial recovery or better. The presented case also had a significant recovery after operation and was discharged with normal limbs strength from hospital and could walk normally without a stick in outpatient follow‐up.

## 4 DISCUSSION

Possible causes of spontaneous SSDH are minor or major spine trauma and nontraumatic causes including anticoagulant therapy, coagulopathy and vascular anomalies (aneurysm, dural arteriovenous fistula (AVF), toxemia of pregnancy, and idiopathic SSDH).^4^, ^5^ Pathophysiology of spontaneous SSDH can be the rupture of the radiculomedullary veins in the subarachnoid space following the trauma or increased intra‐abdominal or intra‐thoracic pressure.^15^ The lesion often develops on the thoracic and lumbar spinal and rarely in the cervical area.^3^ Our patient presented with severe sudden onset cervical pain, left side weakness and Brown Squared symptoms for two days before admission. For diagnosis of spontaneous SDH, MRI is considered the gold standard in evaluation and monitoring of spinal hematomas. Based on post‐hemorrhage period, MRI finding will be changed. Within the first few hours, the clot shows iso‐intensity in T1WI and hyper‐intensity in T2WI. In the first 48 h, hematoma shows hypo‐intensity on T1W1 and hypo‐intensity on T2W1. After 2 days till 1 week, hyper‐intensity and hypo‐intensity in T1WI and T2WI were seen, respectively.^1^, ^21^ In our evaluation, hyper‐intensity on T1‐weighted and hypo‐intensity on T2‐weighted appeared. Based on physical examination and MRI findings, spontaneous SSDH was diagnosed in early subacute stage. Treatment management in previous studies was based on the severity of neurological deficits. Non‐operative treatment may be chosen in patients with minimal neurological deficits, but in those with major deficits, rapid deterioration of clinical and radiological signs and symptoms drainage or surgery should be performed, as we did in our case. Early surgery and aggressive approach is a viable option even in long‐lasting spinal cord compression. It is evident that the outcome mainly varies on the basis of clinical conditions and lesion levels.^1^ In the present study, our patient showed severe neurological deficits and underwent C3‐C7 levels laminectomy; he showed, however, a satisfactory late follow‐up, indicating the short period of time between the onset of the symptoms and the surgical treatment (at most 24 h). It is worth mentioning that those with paraplegia and bladder and bowel dysfunction show lower prognosis, irrespective of conservative or surgical decompression. In adult patients, conservative laminectomy at the cervical surface does not necessarily affect the spinal stability. Based on the authors' knowledge and literature review, our patient was the first C3‐C7 SSDH report treated with C3‐C7 laminectomy. As a result, in such cases, posterior fixation is not performed.

## 5 CONCLUSION

For satisfactory clinical outcomes, urgent clinical and neurological identification with immediate surgical management is required in SSDH cases (at most 24 h after the onset of symptom). Furthermore, extensive operative strategies can be effective and necessary. Cervical laminectomy is predominantly considered as a safe and effective option as long as it is conservative. Also, we should declare that considering the emergency condition of the patient and lack of instrumentation facilities in our center, only laminectomy was done for the patient, although we suggest that laminectomy and fixation would have better results to prevent kyphosis in future.

### AUTHOR'S CONTRIBUTION

The conception and design of the study done by Iman Ahrari, Mohammad Jamali, and Arash Saffarrian. Material preparation and data collection were performed by Reza Taheri, Sulamz Ghahramani, and Mahsa Ghavipisheh. Data analysis was done by Keivan Eghbal and Abdolkarim Rahmanian. The first draft of the manuscript was written by Iman Ahrari and Mahsa Ghavipisheh and Somayeh Moahamadi, and all authors commented on previous versions of the manuscript. All authors read and approved the final manuscript.

## FUNDING INFORMATION

The authors declare that no funding or other support was received during the preparation of this manuscript.

## CONFLICT OF INTEREST STATEMENT

There are no conflicts of interest to declare.

## CONSENT

Written informed consent was obtained from the patient to publish this report in accordance with the journal's patient consent policy.

## Data Availability

The data supporting the findings of current study are available from the Namazi hospital data bank. Also, if needed data would be available from corresponding author upon reasonable request and with permission of Namazi hospital authorities.
